# Participant lived experiences of high-intensity interval training in UK cardiac rehabilitation (HIIT or MISS UK): A qualitative study

**DOI:** 10.1371/journal.pone.0350509

**Published:** 2026-05-29

**Authors:** Charlotte Williams, Richard Neil, Richard Powell, Brian Begg, Stefan Birkett, Simon Nichols, Stuart Ennis, Prithwish Banerjee, Lee Ingle, Rob Shave, Gordon McGregor

**Affiliations:** 1 Edge Hill University, Ormskirk, United Kingdom; 2 Cardiff Metropolitan University, Cardiff, United Kingdom; 3 School of Sport, Exercise and Rehabilitation Sciences, University of Birmingham, Birmingham, England; 4 Cardiff Centre for Exercise & Health, Cardiff Metropolitan University, Cardiff, United Kingdom; 5 Department of Sport and Exercise Sciences, Manchester Metropolitan University Institute of Sport, Manchester, United Kingdom; 6 Centre for Cardiovascular Health Edinburgh Napier University, Edinburgh, United Kingdom; 7 Warwick Clinical Trials Unit, Warwick Medical School, University of Warwick, ‌‌Coventry, United Kingdom; 8 Department of Cardiology, University Hospitals Coventry & Warwickshire NHS Trust, Coventry, United Kingdom; 9 School of Sport, Exercise & Rehabilitation Science, University of Hull, Hull, United Kingdom; 10 Research Innovation Centre, Leeds Teaching Hospitals Trust, ‌‌Leeds, United Kingdom; 11 Centre for Heart Lung and Vascular Health, University of British Columbia-Okanagan, Okanagan, Canada; 12 Department of Cardiopulmonary Rehabilitation, Centre for Exercise & Health, University Hospitals Coventry & Warwickshire NHS Trust, Coventry, United Kingdom; Erzurum Technical University: Erzurum Teknik Universitesi, TÜRKIYE

## Abstract

**Background:**

Exercise programmes are an important component of comprehensive cardiac rehabilitation (CR). High Intensity Interval Training (HIIT) has been proposed as an alternative to conventional moderate intensity steady state (MISS) exercise. In the ‘HIIT or MISS UK’ trial, low-volume HIIT was safe, and clinically and cost effective. However, there is a lack of insight into the lived experiences of those who engage in non-conventional approaches to CR exercise training. The aim of this research was to explore the benefits and challenges associated with HIIT and MISS in CR.

**Materials and methods:**

A qualitative descriptive methodology was adopted to document participant lived experiences. Participants were purposefully recruited from two ‘HIIT or MISS UK’ trial CR centres. After consent, participants took part in semi-structured interviews conducted via Voice over Internet Protocol (VoIP) technologies (e.g., Microsoft Teams (MT), Skype or Zoom). A critical realist approach to inductive thematic analysis was used to analyse the data.

**Findings:**

19 people took part (8 MISS and 11 HIIT; male 18 [95%]; age 59.6 years [SD 10.4]). Analysis revealed a range of perceived psychosocial (e.g., enjoyment, confidence, purpose) and physiological (e.g., weight loss, increased fitness) benefits that were present across both groups (i.e., HIIT and MISS). Participants in both groups identified challenges, for example, a need for exercise to continue beyond what was offered in the trial. There were notable differences across the groups, namely HIIT participants enjoyed feeling challenged, yet grappled with feelings of monotony, whilst MISS participants experienced increased social interaction.

**Conclusion:**

HIIT in CR offered a range of perceived psychosocial and physiological benefits. Integrating more opportunities for social interaction into the HIIT program could enhance participant experience. Practitioners could investigate the feasibility of support for patients after CR has finished.

## Introduction

Cardiovascular disease (CVD) is the leading cause of death globally, with an estimated 19.8 million lives lost in 2022 [1]. In the UK, an estimated 7.6 million people are living with CVD with healthcare costs estimated to be £12 billion each year, and costs to the UK economy equating to approximately £28 billion [2]. Given the magnitude of the CVD burden, it is important to consider how it can be mitigated or managed.

Cardiac rehabilitation (CR) programmes are designed to holistically support those living with CVD. Exercise training is a critical component of CR, conventionally delivered as moderate intensity steady state (MISS) exercise. Research has shown that high-intensity interval training (HIIT) is more effective than MISS in supporting patients to manage CVD [3]. It is time-effective and consists of ‘repeated bouts of relatively hard exercise interspersed with recovery periods of easier exercise or rest’ [4]. The ‘HIIT or MISS UK’ trial [5] reported that cardiorespiratory fitness (VO_2 peak_) improved more in coronary artery disease (CAD) patients who completed an 8-week low-volume HIIT programme (10 x 1 minute high intensity intervals) compared to those completing a MISS programme (1.04 mL.kg^-1^.min^-1^ [95% CI, 0.38 to 1.69; *p* = 0.002]) [6]. The HIIT programme was tolerable and acceptable and cost-effective compared to MISS [7].

‘HIIT or MISS UK’ was a pragmatic trial with interventions delivered in existing NHS CR services. It is important to explore personal experiences to assess the wider psycho-social benefits and considerations for implementation into clinical practice. Research in other populations showed that HIIT, compared to MISS, was well-tolerated, often preferred, and may promote more favourable psychological outcomes, particularly enjoyment and motivation, despite its higher physical demands and lower in-task affect [8–10]. This has not been explored in CR HIIT programmes.

Most research exploring the experiences of HIIT relates to high-volume, body weight and/or sprint interval training protocols, and not in a rehabilitation context. Therefore, the aim of this study was to explore participants lived experiences of low-volume HIIT or MISS programmes in CR by conducting a qualitative study. Specifically, this research focussed on understanding the benefits and challenges associated with low-volume HIIT and MISS CR programmes delivered in the NHS.

## Materials and methods

### Study design

A qualitative descriptive methodology was adopted, whereby a rich description of the experiences of those involved in the HIIT or MISS UK trial was provided. Qualitative description explores a specific phenomenon of interest (i.e., HIIT in CR) through showcasing participant voices [11]. In qualitative description, researchers tend to stay close to the data and focus is placed on analysing words and events as they are presented on the surface from the perspective of the participant [11–13]. However, in any form of qualitative research, there is an element of interpretation. As such, we share our theoretical positioning as this has influenced the way in which we carried out the research. This study is grounded in critical realism, whereby we acknowledge that whilst there is a world independent of human consciousness, the world is socially constructed. That is, the world is made up of individuals’ thoughts, feelings and interpretations which should be explored to obtain an in-depth understanding of reality. It is hoped that the knowledge obtained from the participants experiences in this study can be used to influence future CR interventions [13].

The trial protocol was published previously [5], and the protocol v1.0, dated 1^st^ February 2016, was approved by the NHS Health Research Authority, East Midlands – Leicester South Research Ethics Committee on 4^th^ March 2016 (16/EM/0079). The trial was prospectively registered with ClinicalTrials.gov: NCT02784873

### Participants

Participants were CR patients already enrolled on the ‘HIIT or MISS UK trial’, and purposefully recruited from two CR centres (Atrium Health, Centre for Exercise & Health, Coventry & Ystrad Fawr Hospital, Ystrad Mynach, South Wales). Sampling aimed to be representative of both HIIT and MISS interventions, with the participants involved in both groups invited to take part.

19 participants (male, n = 18) accepted the invitation to be interviewed. Participant data is provided in Table 1. Participants were eligible if they had access to Voice over Internet Protocol (VoIP) technologies (e.g., Microsoft Teams (MT), Skype or Zoom) as online interviews were chosen as the method of data collection. Utilizing VoIP technology enabled interviews to be organized and conducted at a time and place that was convenient for the participant, whilst also ensuring that facial cues and participant behaviour could be observed to support with rapport building.

**Table 1:** Participant characteristics.

	All (n = 19)	HIIT (n = 8)	MISS (n = 11)
Age (mean, SD)	59.4 years (10.2)	56.0 years (11.7)	61.5 years (8.5)
Body mass (mean, SD)	87.7 kg (15.2)	96.0 kg (10.5)	81.6 kg (15.1)
Height (mean, SD)	176.5 cm (6.8)	179.4 cm (6.1)	174.4 cm (6.5)
BMI (mean, SD)	27.8 kg/m² (3.5)	30.0 kg/m² (2.5)	26.7 kg/m² (3.4)
Male	18 (95)	8 (100)	10 (91)
Medication			
Statin/ First line anti-platelet therapy	19 (100)	8 (100)	11 (100)
Beta blocker/ ACE inhibitor/ dual anti-platelet therapy	17 (89)	8 (100)	9 (82)
Initiating Cardiac Event			
Anterior MI	8 (42)	4 (50)	4 (36)
Non-STEMI	6 (32)	3 (38)	3 (27)
Angina	3 (16)	0 (0)	3 (27)
Inferior MI	2 (11)	1 (12)	1 (9)
Treatment			
Percutaneous Coronary Intervention	13 (68)	5 (63)	8 (73)
Coronary Artery Bypass Graft	4 (21)	2 (25)	2 (18)
Medical Management	2 (11)	1 (12)	1 (9)
Comorbid conditions			
Asthma	5 (26)	1 (12)	4 (36)
Osteoarthritis	5 (26)	1 (12)	4 (36)
Musculoskeletal Disorders	4 (21)	2 (25)	2 (18)
History of Cancer	3 (16)	0 (0)	3 (27)
Neurological Disease	2 (11)	0 (0)	2 (18)
Chronic obstructive pulmonary disease	1 (5)	0 (0)	1 (9)
CVD Risk Factors			
Dyslipidaemia	14 (74)	8 (100)	6 (55)
Family history of premature CVD	8 (42)	5 (50)	3 (27)
Hypertension	6 (32)	5 (63)	5 (63)
Excess alcohol	7 (37)	3 (38)	3 (38)

Data as n, % unless otherwise indicated. SD, standard deviation; BMI, body mass index; ACE, angiotensin-converting enzyme; MI, myocardial infarction; STEMI; ST-elevation myocardial infarction; CVD, cardiovascular disease.

### Intervention

A detailed description of interventions (HIIT and MISS) has been published previously [5,6]. In brief, participants attended a CR programme twice per week, for 8 weeks and were randomly allocated to either the HIIT or MISS group for the cardiovascular exercise component of their programme. Participants allocated to HIIT engaged in 1 min intervals on a cycle ergometer (Watt bike), 10 intervals at high intensity (>85% of heart rate maximum) interspersed with 10 intervals at low intensity. For the MISS group, exercise was carried out following the framework provided by the Association for Chartered Physiotherapists in Cardiac Rehabilitation [14]. Cardiovascular exercise consisted of 20–40 minutes of continuous moderate intensity exercise at 40–70% heart rate reserve (HRR) using a treadmill, cycle ergometer, rowing ergometer, and cross trainer. In both programmes, duration and workload were adjusted as tolerated, to adapt to participant progression. Muscular strength and endurance programmes were also completed, whilst participation in a group education programme and home-based exercise was recommended as standard.

### Procedure

Following ethical approval, the lead practitioners at each CR centre made initial contact with participants to invite them to take part in an interview to discuss their experiences of the CR programme. Participants were informed that interviews would explore their experiences prior to, during and after the CR programme. Practitioner A sent a letter out to 125 patients inviting expressions of interest; a follow up call took place two weeks later if they had not received a response. Practitioner B contacted 99 patients via text and email; if they did not receive a response a follow up call took place. Interested participants were identified to the first author.

Forty-nine patients were willing to participate of which 32 (65%) were eligible and invited by email to take part in an online interview between October 2020 and July 2022. Nineteen (59%) accepted (8 MISS and 11 HIIT), 10 (31%) did not respond, two (6%) did not attend arranged interviews, and one (3%) interview could not be organized due to logistical issues.

### Data collection

Semi-structured interviews were adopted as they enable researchers to gain rich, in-depth information regarding participant experiences [15]. Interviews enable researchers to explore participants’ thoughts, feelings, and personal experiences. The utilization of an interview guide provided consistency across each interview, whilst retaining the flexibility needed to explore relevant topics that arose throughout the interviews. Furthermore, the interview guide was designed to encourage open-ended exploration in the responses of participants. It included introductory questions to establish rapport and encourage participants to begin talking about their experiences. The main section of the interview guide explored participants’ thoughts, feelings, and experiences pre, during and after attendance of the HIIT or MISS programme. For example, participants were asked questions such as: “What did you expect the HIIT/ MISS programme to entail?”, “how did you typically feel when you were taking part in each of the sessions?” and “please describe your thoughts of HIIT/ MISS training for CR, now that you have taken part?”. To encourage elaboration, the researcher used follow up questions and probes.

Each interview was conducted by the first author (CW) who has extensive experience. Interviews took place online at a time suitable for the participants, when social interaction was limited due to COVID-19. Prior to the interview commencing, participants were reminded about the research aim, written informed consent was taken, and confidentiality explained. Interviews were conducted between October 2020 and July 2022 and lasted 30–60 minutes.

### Data analysis

Interviews were recorded, and transcribed verbatim. The names of the participants were replaced with ID numbers; all participants who took part in the HIIT programme were allocated an ID number beginning with ‘H’ and all MISS participants were allocated an ID number beginning with ’M’ (e.g., H1 or M1). Thematic Analysis (TA) adopting Braun and Clarke’s (2006) six step approach was used to analyse the data, whilst NVivo 14 was used to organize and manage the data throughout each of the stages of TA [16]. In TA, and in line with Kenny et al. (2024), inductive analysis was chosen as it enabled the research team to make sense of participants lived experience of the CR programme [17]. First, familiarization with the data was conducted through the first author reading and re-reading transcripts. During this, the first author made general notes depicting initial patterns in relation to participants’ thoughts, feelings, and experiences on each transcript. Following this, the first author generated initial codes and grouped similar codes to form ‘subthemes (Table 2). Codes and themes were discussed with the second author as a *critical friend* which encouraged reflection [18]. The first and second authors then reviewed the subthemes against the coded extracts and entire data set. The subthemes were then categorised under the main themes (Table 2). Data saturation was reached with the 19^th^ participant, where we acknowledged that no new themes or insights had been presented.

**Table 2:** Themes and subthemes of the HIIT or MISS UK trial.

Main Themes	Sub Themes
Pre-programme thoughts	Apprehension
	Willingness to do whatever it takes
Psychological Benefits	Enjoyment
	Confidence
	Sense of Purpose
	Relating to Others
Physiological Benefits	Increased Fitness
	Weight Loss
Barriers to the Programme	Work Commitments
	Monotony
	Difficulties with using weights
Post Programme Reflections	Continuation of Exercise Lifestyle Changes Follow up Sessions
Programme Safety	Support from Staff Monitoring
HIIT or MISS	Preference for HIIT
	No preference either way
	Preference for MISS

A member reflections process was adopted, where participants were encouraged to reflect on the research teams’ interpretations of the data, with the aim of providing the participants with an opportunity to confirm or challenge the interpretations and to generate additional data that could enhance our understanding of their experiences. The first researcher shared initial interpretations of the data with participants via email and encouraged participants to consider these and provide feedback. Data obtained from the member reflection process is included in the results.

### Trustworthiness and rigor

To demonstrate trustworthiness and rigor, we adopted Lincoln and Guba’s (1985) four criteria of: credibility, transferability, dependability, and conformability [19]. Reflecting credibility, we adopted member reflections which enabled participants to provide their feedback on our interpretations of the data. Furthermore, through utilizing member reflections we tried to ensure that our findings were reflective of the reality of the participants lived experiences. A reflexive journal was kept in which the lead researcher reflected on their assumptions, biases, and position during key decision-making moments, contributing towards conformability and dependability. Considering transferability, we have provided a thick description of the context in which this research was conducted, and the experiences of those involved in the research. This enables readers to judge the applicability of the findings of this study, to their own contexts.

Elaborating on the reflexive process and acknowledging that personal and professional experiences may influence the analysis, the lead author is a female academic and an accredited Sport and Exercise Psychologist, with experience of conducting qualitative research. Additionally, the lead author has been immersed personally and professionally within the context of physical health rehabilitation, which may have aided the development of rapport with the participants during the interview process. When undertaking the reflexive process, the lead researcher made notes after each interview to explore initial interpretations of the data. These initial interpretations were revisited after each subsequent interview to trace whether interpretations changed as a result of further knowledge about the context and potential biases. Indeed, this process helped the lead author acknowledge any personal biases and became influential in the data analysis process and discussions with the second author.

## Results

Seven themes represent the experiences of 19 participants (8 MISS and 11 HIIT) of the HIIT or MISS programmes. Themes and sub-themes are reflected in Table 2.

### Pre-programme thoughts

Participants were asked to discuss their thoughts regarding HIIT and MISS programmes prior to starting. Two sub-themes emerged in the broader theme of pre-programme thoughts, *apprehension,* and a *willingness to do whatever it takes*.

#### 3.1.1. Apprehension.

Some participants in the HIIT group experienced pre-programme concerns, reflecting participants’ *apprehension* regarding their ability to engage in high-intensity exercise after a cardiac event. Participant H4 explained that they felt “vulnerable” after their cardiac event, and they were unsure of what their heart could withstand:

She [the practitioner] says it’s high intensity training, and I said, ‘yeah but I had a heart attack 3-4 days ago… really?’ Because at this time I was all over the shop. I didn’t know if I could even lift a cup of tea… I felt weak and vulnerable. I’m thinking, ‘I’ve just had a heart attack, it’s going to put increased stress on the heart. Is that good idea?’

Other participants in the HIIT group questioned their physical ability due to being unsure of what they were physically capable of, as reflected by participant H8:

Immediately after the surgery, my thought process [is], ‘physically am I able to do [the exercise]?’ The feeling myself is that I was not able to do this. So, I am not able to lift such heavy, not heavy, even 10K or 20K bags.

Some participants in the MISS group also aired their concerns regarding their ability to exercise. Participant M10 stated:

The first session you are a little unsure because you’re suddenly asking your body to do things that you know you’re not sure of when you have just had this operation. It makes you [think], ‘I better not do any too stretchy exercises,’ because after the operation you were told you were not to put any weight down on your hands to push yourself out of the chair and to pick up heavy things.

Participant H3 commented on the apprehension they experienced after being placed in the HIIT group:

It was daunting at first, what they were expecting me to do because after me just having a heart attack I was thinking ‘what’s going on here, I am in the high group’ … but I went with it and I was happy to do it.

#### 3.1.2. Willingness to do whatever it takes.

Whilst some participants experienced apprehension, other participants showed a *willingness to do whatever it takes*. Participants were grateful to be part of the CR programme, with some participants expressing gratitude and feeling that they had been given a second chance. As such, some participants were willing to take part in whatever they needed to do to help themselves recover. For example, participant H2 stated: “I thought, it had to be done. [After] what had happened, I thought, well…I’ve got to change my lifestyle anyway. So do it then”. In addition, participant M5 stated, “The reason I’ve been referred to this programme is for my benefit. I’m lucky to be on it and that’s something I really appreciated”.

#### 3.1.3. Psychological benefits.

Four sub-themes emerged in the broader theme of psychological benefits: *enjoyment, confidence, sense of purpose* and *relating to others*.

### Enjoyment

Participants in the HIIT and MISS groups expressed a general sense of *enjoyment* in taking part. For example, participant H8 stated “it was an enjoyable experience”, and participant H2 commented, “I found it where it was quite enjoyable to get back into something where I haven’t done anything for such a long time.” Participant M8 stated, “I thoroughly enjoyed myself and it was something I can recommend to anybody”. Furthermore, participant H4 commented, “the more I did, the more I enjoyed it, I ended up doing an hour and 20 each session”.

Exploring the reasons behind enjoyment of the programme, some participants associated their enjoyment with the challenge of exercise. This was particularly evident with the HIIT participants. For example, participant H1 described enjoying the “strenuous” exercise and stated that they “enjoyed the feeling of being worn out”. Adding to this, participant H3 explained that the HIIT programme felt familiar, that they enjoyed the challenge, stating, “I know what it is like to go the distance and push yourself to the limits. I enjoyed it.”. One participant in the MISS group associated their enjoyment of the programme with experiencing challenge, “I used to enjoy the challenge, to the point where on the treadmill I quite quickly got to a stage where I was on the highest incline you could get on and working quite hard in terms of speed.”

Participants also discussed experiencing enjoyment from knowing that they were doing something that was good for them. Participants felt that the HIIT training was generally beneficial. Participant H5 explained, “I enjoyed the exercises…it was something that I knew would be beneficial to me”. Additionally, participant M2 said, “I think I enjoyed the whole session, to be honest, because I could feel the benefit of it quite quickly”. Participant H7 reflected on enjoying seeing the changes that they were making in themselves, “[I] actually felt I was making a difference in myself, that I was making an improvement from a health perspective and a fitness perspective”.

#### Confidence.

Participants described an increase in *confidence* in their ability to carry out everyday tasks. Whilst participants experienced pre-programme apprehension associated with an inability to carry out day to day activities, both the HIIT and the MISS programmes instilled a newfound confidence in their ability to engage in day-to-day tasks. Participant H4 explained:

It was all about confidence… doing those exercises, the more I knew I could do in the gym, the more I knew I could lift things at home. In my head, it told me I can do things. I’ve gone from, I don’t know if I can lift a cup of tea without worrying about it to lifting the dogs’ big parcel of food, which is 20-25kg… and moving it with no thought about it.

Participant H5 described gaining confidence from discovering their own personal tolerance levels:

[my] confidence on what activities I could undertake was quite low after the heart attack, this [HIIT] built it back up… it tested me, it stretched me, and it’s given me some parameters as to what I can do and what I can’t. Because, when you’ve had a heart attack, you need to explore in terms of what is tolerable for you to do and what isn’t. And this pushed me a bit, to a level where I can do my normal day-to-day life.

In addition, participant M6 shared their experience of gaining confidence as a result of the programme:

[the programme was] very beneficial, primarily in terms of giving me the confidence to know that I could push the body, and the body would respond and would cope with it. I think that was a benefit that came out of the exercise programme that I felt confident to be able to push it more than I used to before it all happened.

Participant M2 discussed a change in their attitude from increased confidence levels, stating, “[I was] encouraged to go as far as I can and that really was a big confidence builder, my attitude then changed to ‘*well yes actually I can… I can actually push myself and do what I did before’”*.

#### Sense of purpose.

Some participants expressed a renewed *sense of purpose*. Participants shared insight into the difficulties that they faced after their cardiac event that often prevented them from engaging in day-to-day activities. However, participants highlighted that the HIIT and MISS programmes gave them a sense of purpose, and something to look forward to. Participant H8 stated: “not being in work anymore, it [HIIT] was something to [look forward to]”. Additionally, participant H3 commented: “it was something to look forward to because I was off [work] for about three months, it just gave me a goal, something to do”. Participant M1 elaborated on these comments, providing insight into how the programme acted as a replacement for work:

It actually gave my week purpose, it was almost like going to work, you had something to aim for that couple of times a week to go and do that, you know I suppose it is just that word purpose, it just gave you something to focus on.

#### Relating to others.

Participants predominantly in the MISS group shared their positive experiences of getting to know other participants and being able to relate to others in their group. Participant M3 stated, “it was nice in some ways to be in a group of people who had suffered some sort of disability or illness from heart trouble, I did make a few friends or acquaintances, who you chat to every day that you were there and got to know”. One participant explained that the benefits associated with the programme were in part down to the exercise itself, but also as a result of being around people who had also experienced cardiac events. Participant M8 said:

The positive of it was because you were with other people that were the same, so 50% is the exercise and the monitoring and making sure you’re safe but at least 50% is being with people that the same thing has happened to.

### Physiological benefits

Two sub-themes emerged in the broader theme of physiological benefits: *increased fitness* and *weight loss*.

#### Increased fitness.

Participants in the HIIT and MISS programmes believed that their *fitness increased* as a result of taking part in the exercise programme. Indeed, participants advised that they felt their *general* level of fitness increased. Participant H1 stated, “[I’m] getting fitter and healthier… Walking up the stairs is easier, there’s nothing better than that”. Additionally, participant M9 shared their experience of the changes to their fitness stating,

I did get fitter, I noticed erm a bit of a difference, you felt you were getting a bit fitter, I did notice my arm muscles were very slightly bigger than they were when I started, you know, I mean I think I felt noticeably fitter at the end of it than I did at the start.

Participant H8 noted that their friends used to always complain about how slow the participant was when they went out for walks, now they stated, “I am able to run fast, work fast… and now my friends are not complaining”. Furthermore, participants expressed that the more they exercised, the easier the exercise became. For example. Participant H4 stated: “I felt I was getting fitter; I felt the more I did the fitter I got… it’s like my breathing felt better, I’m a bit asthmatic and I can get a bit croaky, so it was helping with that”. Other participants described experiencing quicker recovery times between exercises. Participant H7 stated:

Another really good bit was when you feel yourself that it doesn’t take you that full minute to recover towards the end, you’ve got like 30 seconds and then you go, *ah I feel okay now*. When you see that sort of time increment getting longer that feels really good as well.

#### Weight loss.

Over half of the HIIT participants and half of the MISS participants shared that they felt they had *lost weight* as a result of the programme. Participant H1 stated that they were sure they had lost weight as a result of the programme due to ‘their clothes fitting them better’. Additionally, participant H6 stated, “I’ve lost weight. I’ve lost a stone”. Additionally, participant M5 shared their weight loss experience, “Over the period of the two months, I lost about a stone, and I’d never been physically better. I felt good”

### Barriers to the HIIT/ MISS programmes

Three sub-themes emerged in the broader theme of barriers to the programme: *work commitments*, *monotony,* and *difficulty with using weights*.

#### Work commitments.

Participants in the HIIT and MISS groups discussed potential barriers associated with work commitments. Participants explained that on some occasions the timing of the exercise classes either clashed with work meetings, or they had to squeeze classes around meetings and other work commitments. Participant H7 explained, “getting there sometimes during work hours was probably the most challenging bit”. In addition, participant H1 noted that any absence from the sessions was usually down to work commitments, “If I didn’t go it was because I’d have to do work”. Work was also noted as a potential barrier for participants in the MISS group, with participant M2 explaining:

At the time [I was] self-employed so I had commitments, so actually getting to the sessions was a bit of a task, to put time aside to get [there], because erm I couldn’t have just turned up from my working environment into the gym environment, you know especially with dust and sawdust all over me. It entailed going and having a shower and getting changed and going to the gym and then coming from the gym and going back to work again… So, it was… it was an effort getting there.

#### Monotony.

Participant H6 in the HIIT programme highlighted some sessions became “*monotonous*” and discussed the need for more variety to the exercises in the HIIT programme stating:

To be honest, after the 16th sessions doing the same exercise, it was becoming a bit monotonous and that’s because you understand what you had to do, and it was just the same exercise. Maybe if it wasn’t on the bike. Maybe if you did HIIT on the bike on Monday and come Thursday you did HIIT on the cross train or something.

Participant H8 also discussed being drawn more to a variety of exercises than just one (i.e., the bike). In comparing the two CR exercise programmes, they stated:

The circuit type training was perhaps more acceptable than just being on the bike, you know if I had the choice [of] either you’re going to sit on the bike for half an hour or you can go around doing the circuit training, then I would have picked the circuit training… but having said that erm that is the only advantage I could see.

#### Difficulties with using weights.

Some MISS participants indicated that they found lifting weights difficult, as Participant M10 acknowledged, “I think some of the weight things were more challenging”. Participant M4 did not enjoy the weights side of the MISS programme at all, stating, “Heavy weights… I hated doing that, so I stopped doing that”. Participant M3 explained the physical impact lifting weights was having on them, “The weights were a little bit difficult, mainly because I was starting to get muscular skeletal problems with it more than breathlessness or anything, I could manage the weights but there was the suffering afterwards”.

### Post programme reflections

Three sub themes reflect the broader theme of post programme reflections, *continuation of exercise*, *lifestyle changes*, and *follow up sessions.*

#### Continuation of exercise.

Six of the nine HIIT participants *continued to exercise* after attendance at the CR exercise programme. Of these, four continued with HIIT at their local gym. Participant H7 described their engagement in exercise after the rehabilitation programme, “I go back down to the same gym now and I still do high intensity on the bike”. Additionally, participant H4 stated, “I’m still doing the HIIT stuff, I go to the gym twice a week and I’ll try to do 15-20 minutes on the bike doing the HIIT. I’m building back up because of the lock down”.

Two of the six participants engaged in running, walking and/or cycling after the HIIT programme. Participant H1 continued their exercise journey and used the HIIT programme as a starting point for continued engagement in exercise:

I kept up my fitness regime… I would go out in the garden, and I would do exercise in the garden from all the group sessions I could remember to get my heart rate up because I remembered that it’s important. Get your heart rate up. Then I’d go for a run, jog. And for a long time, that was really great… I started running and I started enjoying running. And I ended up doing park runs. Then we made it a bit of a thing if we go on holiday in the UK, we find out where there are park runs and take part.

Eight of the ten MISS participants *continued to exercise* after the MISS programme. Of these, two began HIIT training, whilst the remainder engaged in walking and circuit style training. Participant M3 explained that they started HIIT training because the exercise they were doing wasn’t increasing their heart rate enough due to their increased fitness:

I have got a HIIT programme that I do, HIIT exercises, mainly because I got fitter to the point that I had to really struggle to get my heart rate up… I got to the point now where I really have got to work hard to get it up over 80 [bpm].

Participant M7 explained how they continued exercising in a similar way to the way they were exercising on the MISS programme, “I did continue it, I was determined to continue it… I didn’t mind paying, and my‌‌ local council have one, so I started on my own with a local council one, similar sort of thing”.

#### Lifestyle changes.

Some participants in the HIIT CR programme experienced *lifestyle changes*, whereby exercise, and healthy eating became part of their everyday routines. Some participants had low levels of exposure to healthy eating and/ or exercise pre-CR, however the HIIT programme seemed to kick start their exercise journeys and subsequent lifestyle changes. Participant H1 illustrated this in the following quote:

Well, this [HIIT] was central to my health. I used to drink a lot, smoke a lot. I’ve Given all that up and. I was sleeping better. Whereas before I would hardly ever sleep. I was sleeping a lot better. I think I felt I was eating better as well. You know, so it [HIIT] has done a lot. It did change sort of like a big lifestyle change… It [HIIT] got me on track for sort of getting healthier. Took my mind off stuff like not wanting to go to the pub. Not wanting to smoke.

In addition, participant H1 reflected on the changes they made to their lifestyle after CR:

I was a desk jockey and a couch potato, but I’m definitely more mobile. In fact, I’ve got to the point where I don’t want to sit on the sofa anymore. I want to be up and about, and I find myself moving around, getting things done. I’m actually really keen on being up and about. I was at the peak of fitness in my 50s, which hadn’t happened since I was in my early 20s. I was enjoying doing exercise. I was more confident at work. I was more confident in social life. And I was generally more mobile.

#### Follow-up sessions.

Participants in both the HIIT and MISS programmes shared their desire for there to be *follow up sessions* with practitioners at the CR centres. Participants were “disappointed” that the programme had come to an end and found it difficult to motivate themselves without the structure of formal sessions. Participant H1 highlights this:

It’s then down to your own motivation and then work gets in the way, life gets in the way. Not this week. I’ll do it tomorrow. I’ll leave it for a week or weather’s not nice this week. Or it’s too hot, it’s too rainy. And then gradually you find you go months and you haven’t done it.

To address these issues, participants suggested that there could be scheduled follow up sessions. Participant M10 stated:

I would have liked a follow up perhaps 6-12 months later to encourage continued supervised exercise for another limited period of time. Motivation reduced after the supervised sessions ended plus covid got in the way.

Participant H8 added, “It would be great if I get a chance to be reassessed by the NHS nurse in the rehab centre, even though I am not going there regularly”. Participant H1 suggested a spin off approach to the programme whereby former patients could attend scheduled sessions, “Perhaps an annual programme could be set up for former patients to attend an exercise programme of say 2-3 months to keep up the good work”.

### Perceptions of programme safety

All participants perceived the HIIT and MISS programme to be appropriate and safe. Two sub-themes emerged in the main theme of programme safety, *support from staff* and *monitoring.*

#### Support from staff.

Participants discussed safety in relation to receiving *support from the staff* involved in the programme. Indeed, it was evident that participants trusted the professional staff members that were responsible for delivering the HIIT programme. As a result of the trust in staff, participants believed they were well looked after and that the programme was safe. This is illustrated when participant H7 stated, “I knew I was in safe hands… the guys who ran it, they’re great people so they weren’t going to put you in harm’s way or anything”. Participant H3 expands on this by stating, “I had my doctor there, plus I had my A&E local, so I was happy to continue and obviously the medical people at the actual gym were more than qualified”.

Participant M5 spoke highly of all staff involved in relation to safety and support:

These guys are professionals, and you know they’ve got your back. I never thought for a minute anything would happen to me, but I was reassured to think if I do feel unwell at any point, I’m in no better place. The support and the intervention, it felt they were just right, you know. There’s enough staff around that you can tap somebody on the shoulder if you want to have a chat with somebody about something.

Participants seemed to take comfort in knowing that the staff running the HIIT programme would only ask participants to complete exercises or increase intensity to a level which they knew participants could manage. This is reflected by participant H5 when they stated, “I took the view that I wouldn’t be asked to do anything that the trainers considered outside or inappropriate, so I was comfortable”.

#### Monitoring.

Participants sought comfort in being observed by staff whilst they were taking part in the exercise. This form of *monitoring*, alongside the monitoring of physical data (e.g., heart rate) allowed participants to push themselves. For example, participant H4 stated, “I knew that I could try a bit harder because it was in a safe place to do it”.

In addition to monitoring encouraging participants to push themselves, it also gave participants a sense of comfort in relation to their capability and safety. Participant H5 stated, “I wasn’t being asked to do anything that I wasn’t considered capable of handling. I felt very comfortable that what I was doing was being monitored”. In addition, participant M9 stated:

You have got the people there that are keeping an eye on you, you have your blood pressure taken, you have got heart rate monitors to start with, so it is not as if you’re there by yourself. You know you have got people who are you know clinically trained, so it felt a very safe erm environment.

### HIIT or MISS?

Each participant was asked if they had the choice, would they have preferred to be a part of the HIIT or MISS programme.

#### Preference for HIIT.

Twelve participants (8 HIIT, 4 MISS) stated they would choose the HIIT programme over the MISS programme. Participant H4 stated, “I’m really glad I did it, and I would recommend it to anybody”. Whilst participant H4 commented on how they enjoyed the ‘*challenge*’ of the HIIT programme:

I think the HIIT programme scores higher because it pushes you on. Because it boils down to how vulnerable you feel after a heart attack. Do you get pushed by an ordinary gym session to the same extent? I don’t think you do. And you certainly don’t get that spike in heart rate unless you push yourself. You’re in a place where you don’t want to push yourself because you don’t really want to do any damage. So yeah, HIIT programme for me every time.

Participant M6 agreed with welcoming a challenge stating, “looking at what they [HIIT participants] were doing I thought hmm, that is a challenge and yes I would have relished the challenge”.

Some participants in the MISS group chose the HIIT programme as they were ‘*curious’* to explore a different type of training. Participant M8 stated, “High, the high intensity. Just because curiosity really, I want to see if I can do it”. Participant M9 agreed, “I would choose the HIIT one because I would be curious to see what difference that actually made, having done the MISS part, you know I would want to see what difference.”

#### No preference either way.

Five participants (5 MISS) stated that they would have engaged in either HIIT or MISS CR programmes. The dominant reason was that they were happy to do any of the exercise programmes as they were in a controlled environment where they felt they were being supported. Participant M10 stated, “I think the two of them are as good as each other to be honest. It’s down to the needs of the individual person”.

Participants explained how important it was to be monitored when exercising after undergoing major heart surgery. As such, it did not matter to the participants which programme they were engaged in, as long as they were supported and being monitored by staff. Participant M8 stated:

When you’re offered something like that [exercise programme], no matter what it is called, you want to do it because it gives you that comfort blanket… That there is somebody there looking at you while you’re exercising that is going to tell you if you’re OK, it is a sort of a… a comfort blanket, isn’t it?

#### Preference for MISS.

Two participants (1 HIIT, 1 MISS) stated that they would rather have done MISS. Participant M4 did not give a reason for this, stating “I would prefer the MISS group”. Whilst participant H8 explained that they would have preferred the variety of exercises available in the MISS programme:

To have more of a variety I would like to go for that one [MISS]… I suppose given the opportunity if you had said you can either go on the bike or you can do these circuit exercises, I might have gone for the circuit exercises but at the time it never bothered me.

### Discussion

The HIIT or MISS UK trial demonstrated the positive physiological and health economic benefits associated with HIIT, compared with MISS, for patients attending CR in the UK. The purpose of the present qualitative research was to illustrate patients’ experiences of engaging in a HIIT or MISS programme, exploring the associated benefits and challenges.

Participants discussed several perceived psychosocial benefits from taking part in the CR exercise programmes. HIIT participants enjoyed the programme and believed that their mood improved as a result. This aligns with scoping and systematic reviews that recognised enjoyment as a positive psychological outcome associated with HIIT [8–10]. This is also reported with HIIT for those living with obesity [20] and recreational runners [21]. The enjoyment of HIIT has been attributed to the variation in exercise protocol characteristics, to HIIT being less time consuming, and to an increase in motivation due to faster and greater benefits [22]. Our findings support the attribution of increased motivation, with HIIT participants enjoying challenge posed by HIIT, which was motivating. Participants enjoyed how much HITT pushed them to work outside their comfort zones. Some participants, however, reported monotony due to being on one apparatus (Wattbike) for the entire session. To enhance enjoyment, practitioners could consider integrating more variety in the exercises and/or apparatus used. There is no reason why HIIT cannot be prescribed using a variety of exercise modalities rather than a single apparatus. However, more supervision and monitoring would be required.

Consistent with findings from Way et al. (2020), participants in both groups reported that confidence to undertake CR exercise and activities of daily living increased [23], such as unloading the shopping from the car. Although this was not unique to either HIIT or MISS, it is an important finding given that patients who experience cardiac events often lose confidence in engaging in day-to-day activities, and experience a lack of autonomy.

Participants in the MISS group discussed the importance of being involved in a group and the comfort they experienced being around people they could relate to. This is well documented in the CR setting [24]. In contrast, relating to others and social support was not discussed by HIIT participants. This may reflect the way in which the HIIT programme was designed and delivered, as individuals generally exercised in isolation. If connecting with others is a key mechanism for participants to adhere to exercise programmes during and after CR, it may be beneficial for HIIT programmes to be designed to encourage social interaction.

Fourteen participants continued to exercise after CR. Whilst the reasons for exercise continuation was not a focus of this evaluation, some of the responses suggested that enjoying exercise and understanding the benefit of ‘pushing themselves’ could influence maintenance. One reason a MISS participant gave for wanting to engage in HIIT after CR was to increase the challenge, which suggests an increase in the belief in their ability to exercise. These findings support existing work [25] in which the belief in exercise capability was a key driver in exercise maintenance after CR.

Some participants did not engage in exercise after CR. Work and life commitments were key barriers. However, it is important to acknowledge wider issues that may have influenced this, e.g., some participants were living through the COVID-19 pandemic. Exercise at external locations became limited which may have interrupted the ‘flow’ of exercise [26]. Nevertheless, if work and life commitments are potential barriers, then CR programmes could be complemented by lifestyle management sessions to support exercise being integrated into daily routines after CR.

Some research has questioned the safety of HIIT programmes in CR. In the current study, some participants initially experienced concerns regarding the safety of HIIT after a cardiac event. However, on completion of the programme, all participants perceived HIIT to be safe and appropriate. This is supported by empirical data demonstrating the safety of HIIT in CR settings [6,27]. Participants in the HIIT programme perceived they were safe because they felt supported by the staff supervising the sessions and believed that they would not put them under any pressure that they could not withstand.

### Implications for practice

HIIT was perceived as a safe and appropriate CR programme that could be adopted in practice. Adding to the clinical data from the HIIT or MISS UK trial, adopting HIIT in CR settings offers a range of perceived psychosocial and physical benefits. A key challenge to implementation identified by participants, which is also prevalent in wider literature, was the lack of follow-up and support after the programme had finished [25]. Researchers and practitioners should consider supporting patients beyond their ‘official’ CR programme alongside support to integrate exercise into everyday life, and maintain social connection. Practitioners could also consider organized support groups or community initiatives to encourage prolonged engagement in exercise.

### Limitations

The time between participants completing the intervention and the interviews being conducted was greater than anticipated. As such, reflections on experiences may have been distorted based on participants current knowledge, beliefs, and expectations [28]. As a collective, participants that accepted the offer of an interview were thankful for the rehabilitation offered and, therefore, may have provided responses that they believed would support the programme. The research team attempted to minimize social desirability through ensuring privacy and communicating anonymity procedures. Furthermore, if the interviewer suspected socially desirable responses, they used indirect questions and probes for more information [29]. Whilst we aimed to minimize bias, the research team acknowledge the potential for participation bias potentially leading to positively skewed outcomes. While barriers to participation were identified (more negative outcomes) and explained by those interviewed, those who did not volunteer to participate may have offered different experiences and insights. Further, 18 of 19 participants were male, limiting the generalizability of our findings. The requirement of VoIP technology for interviews may have systematically excluded older or socio-economically disadvantaged patients.

### Future research

It would be valuable to explore follow up programmes after CR. That is, what motivates participants to continue exercising after CR, what deters participants, and does this differ based on the type of exercise (e.g., HIIT/ MISS). The perceptions and experiences of intervention practitioners would also be insightful.

## Conclusion

Participating in either HIIT or MISS CR provided perceived psychological and physiological benefits, underpinned by the physical challenge of HIIT and the social connection of MISS. Work and life-related commitments were potential barriers to continuing exercise after CR. As such, it would be beneficial to explore how CR programmes can support individuals once the programme has come to an end.
